# Epidemiological and etiological investigations of hand, foot, and mouth disease in Jiashan, northeastern Zhejiang Province, China, during 2016 to 2022

**DOI:** 10.3389/fpubh.2024.1377861

**Published:** 2024-05-01

**Authors:** Yongjuan Yuan, Yun Chen, Jian Huang, Xiaoxia Bao, Wei Shen, Yi Sun, Haiyan Mao

**Affiliations:** ^1^Jiashan County Center for Disease Control and Prevention, Jiaxing, Zhejiang, China; ^2^Zhejiang Provincial Center for Disease Control and Prevention, Hangzhou, Zhejiang, China

**Keywords:** hand, foot, and mouth disease, human enteroviruses, molecular epidemiology, co-circulation, VP1 region, phylogenetic tree

## Abstract

**Background:**

Hand, foot, and mouth disease (HFMD) is a common infectious disease in children. Enterovirus A71 (EV71) and coxsackievirus A16 (CA16) have been identified as the predominant pathogens for several decades. In recent years, coxsackievirus A6 (CA6) and coxsackievirus A10 (CA10) have played increasingly important roles in a series of HFMD outbreaks. We performed a retrospective analysis of the epidemiology of HFMD and the spectrum of different viral serotypes, to elucidate the genetic and phylogenetic characteristics of the main serotypes in the Jiashan area during 2016 to 2022.

**Methods:**

Descriptive epidemiological methods were used to analyze the time and population distribution of HFMD in Jiashan during 2016 to 2022 based on surveillance data. Molecular diagnostic methods were performed to identify the viral serotypes and etiological characteristics of HFMD. Phylogenetic analyses was based on VP1 region of CA16 and CA6.

**Results:**

The average annual incidence rate of HFMD fluctuated from 2016 to 2022. Children aged 1–5 years accounted for 81.65% of cases and boys were more frequently affected than girls. Except when HFMD was affected by the COVID-19 epidemic in 2020 and 2022, epidemics usually peak in June to July, followed by a small secondary peak from October to December and a decline in February. Urban areas had a high average incidence and rural areas had the lowest. Among 560 sample collected in Jiashan, 472 (84.29%) were positive for enterovirus. The most frequently identified serotypes were CA6 (296, 52.86%), CA16 (102, 18.21%), EV71 (16, 2.86%), CA10 (14, 2.50%) and other enteroviruses (44, 7.86%). There were 71 and 142 VP1 sequences from CA16 and CA6, respectively. Substitution of N218D, A220L and V251I was detected in CA16 and may have been related to viral infectivity. Phylogenetic analysis showed that CA16 could be assigned to two genogroups, B1a and B1b, while all the CA6 sequences belonged to the D3a genogroup.

**Conclusion:**

CA6 and CA16 were the two major serotypes of enteroviruses circulating in the Jiashan area during 2016 to 2022. Continuous and comprehensive surveillance for HFMD is needed to better understand and evaluate the prevalence and evolution of the associated pathogens.

## Introduction

Hand, foot, and mouth disease (HFMD) is a common infectious disease of children caused by an extensive spectrum of human enteroviruses (HEVs) (1). Most patients have mild symptoms; however, a small number have aseptic meningitis, encephalitis and acute flaccid paralysis, and some critically ill children show rapid disease progression and may die (2, 3). HFMD has caused significant medical, social, and economic burdens to the global community, and has become a major public health problem worldwide, especially in East and Southeast Asia (4, 5). In China, HFMD outbreaks have occurred annually in the past decade. A nationwide surveillance system was launched in 2008 in which HFMD was categorized as a class C notifiable disease.

HEVs are nonenveloped, single-stranded, positive-sense RNA viruses (6). HEVs comprise more than 100 serotypes. Enterovirus A71 (EV71) and coxsackievirus A16 (CA16) have historically been regarded as the most common pathogens responsible for HFMD outbreaks in China (7, 8). A newly emerged enterovirus, coxsackievirus A6 (CA6), has rapidly replaced EV71 and CA16 as the predominate pathogen causing HFMD in mainland China since 2012 (9). HFMD cases associated with coxsackievirus A10 (CA10) have increased in most regions, especially since EV71 vaccination programs began in China during 2016 (10). The rapid change in pathogen spectrum constitutes a new challenge to the prevention and control of HFMD.

A molecular typing system based on enterovirus VP1 was established because VP1 sequences correlate well with antigenic typing and identification (6, 11). A surveillance system for HEV was established during 2002 in Zhejiang Province (12). Therefore, both sporadic and outbreak cases of HFMD caused by HEVs had been reported in Zhejiang Province for several decades (13). Jiashan County is located in the northeast part of Zhejiang, at the center of China’s Yangtze River Delta, near Jiangsu Province and Shanghai Municipality. Although HFMD cases have increased dramatically, systematic research on epidemiological profiles and genetic characteristics of HFMD is limited in this area, as in other small counties in the Yangtze River Delta. To address this problem, we investigated the epidemiology of HFMD and the diversity of viral serotypes in Jiashan during 2016 to 2022. We also elucidated the genetic and phylogenetic characteristics of the main viral serotypes isolated in that period, based on the VP1 region. Understanding these features will be crucial for the development of a long-term strategy at community level to reduce the burden of associated disease and prevent sporadic cases and outbreaks of HFMD. Continued surveillance of the changing epidemiologic pattern of HFMD will inform the formulation and targeting of candidate polyvalent vaccines and antiviral therapies.

## Materials and methods

### Samples

Sentinel pediatricians were asked to collect clinical specimens from patients presenting with HFMD and to report cases to the surveillance system according to the national guidelines for control and prevention of HFMD (10). Specimens were sent routinely to the virology laboratory at Jiashan Center for Disease Control and Prevention (CDC) for detection of HEVs.

### Laboratory work

HEV RNA extraction, RT-PCR identification and VP1 gene sequencing were performed as described previously (13). Geneious Prime^1^ was used to assemble VP1 sequences. BLAST was used to identify the different viral serotypes. All sequences obtained were deposited in GenBank (OR780127–OR780339) (Supplementary Table S1). Other sequences used in the phylogenetic trees were downloaded from GenBank and alignments were performed. The phylogenetic tree was constructed as described previously (12). The patients’ data, including sex, age, place of residence and onset of illness, were collected from the Chinese Information System for Disease Control and Prevention. The general epidemiological characteristics of the demographic information were presented through descriptive analysis. Categorical variables were displayed as counts and proportions. *T*-tests were utilized for continuous variables and chi-square tests for categorical variables. EpiData 3.0 software^2^ was used to establish the database. SPSS 26.0 (SPSS Inc., Chicago, IL, United States) was used for statistical analysis. *p* < 0.05 was considered significant.

## Results

**Figure 1:**
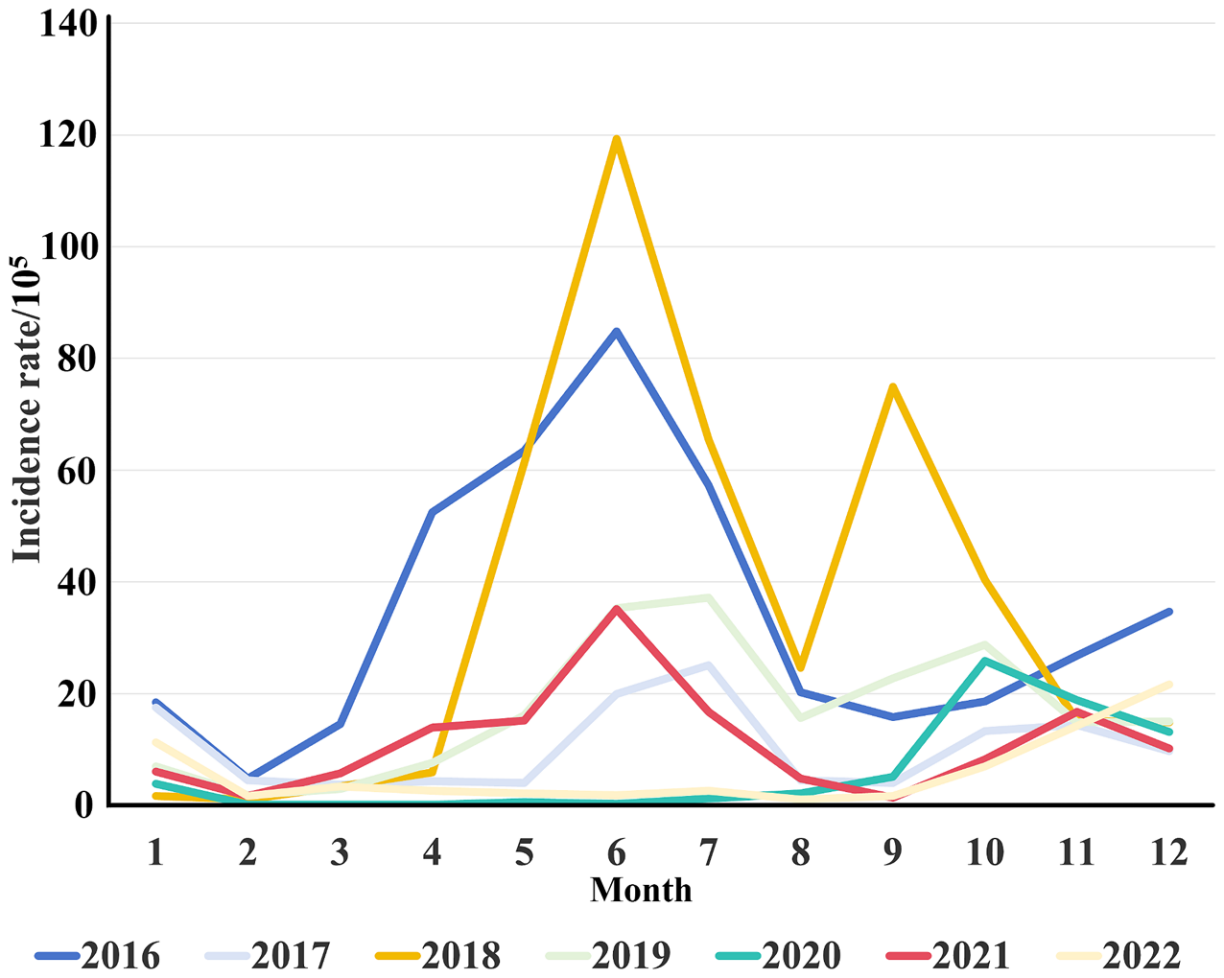
Monthly distribution of HFMD incidence from 2016 to 2022. The different colors represent the monthly incidence rates per 10^5^ population of HFMD across different years.

**Figure 2:**
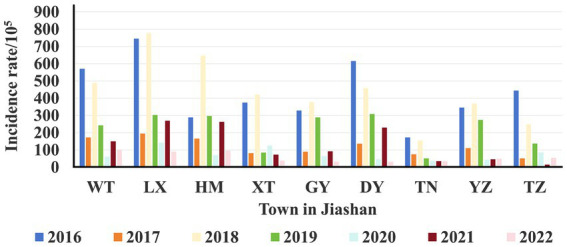
Area distribution of HFMD incidence from 2016 to 2022. The different colors represent the incidence rates per 10^5^ population of each township across different years.

A total of 24,559 clinical cases of HFMD were reported in Jiashan during 2016 to 2022. The average annual HFMD incidence rate was 585.25 per 100,000 population (range: 391.72–804.31). The incidence rate decreased from 2016 to 2017, increased from 2017 to 2018, decreased from 2018 to 2021 and finally increased to 2022 (Table 1). Among these HFMD cases, 14,602 were in males and 9,957 in females, with a ratio of 1.46 (range: 1.34–1.53). The average incidence rate in males was 255.44 per 100,000 population, while in females, the rate was 193.88 per 100,000 population, which was a statistically significant difference (χ^2^ = 8.306, *p* < 0.01). Patients were mainly aged 1–5 years, which accounted for 81.65% of cases. Except for 2020 and 2022, the number of cases in other years peaked from June to July each year, followed by a small secondary peak from October to December and decline in February. The number of cases in 2020 and 2022 was different and increased slowly (Figure 1). The urban district of LX had a high average incidence rate (360.27 per 100,000), while the rural area of TN showed the lowest average incidence rate (79.71 per 100,000) during 2016 to 2022 (Figure 2).

**Table 1:** Distribution of reported and detected HFMD cases in Jiashan County from 2016 to 2022.

Variable	2016	2017	2018	2019	2020	2021	2022
HFMD Reported Cases	4,564	3,232	4,636	3,869	2,521	2,539	3,198
Incidence rate/10^5^	775.20	545.51	804.31	659.22	425.84	391.72	494.94
**Gender**							
Male (Incidence rate/10^5^)	511.03	150.13	554.76	255.46	86.56	146.99	83.12
Female (Incidence rate/10^5^)	390.05	121.98	401.33	194.56	69.47	121.96	57.84
Gender ratio	1.52	1.51	1.53	1.35	1.34	1.51	1.47
**Age Group (Incidence rate/10^5^)**							
0-	3087.75	1619.83	3843.11	1252.35	388.62	855.86	374.63
1-	17010.56	5009.59	18175.18	4476.50	2027.37	2886.63	1632.16
2-	11876.48	2965.60	12208.04	5647.35	1071.95	2015.19	1042.97
3-	13777.42	3553.30	13937.09	5631.07	2009.59	3465.56	2097.63
4-	8794.87	3073.77	9433.47	3461.70	1648.22	3124.30	1186.03
5-	4480.04	1166.26	5312.41	2413.15	733.82	1519.31	972.25
**Specimens**							
EV-positive (%)	95.16%	93.55%	83.72%	90.54%	73.81%	82.47%	77.89%
EV-A71 (%)	9.68%	14.52%	0.00%	0.00%	0.00%	1.03%	0.00%
CVA16 (%)	22.58%	4.84%	34.88%	32.43%	1.19%	15.46%	15.79%
CVA6 (%)	53.23%	61.29%	31.40%	48.65%	58.33%	60.82%	56.84%
CVA10 (%)	1.61%	6.45%	5.81%	0.00%	3.57%	1.03%	0.00%
Other EVs (%)	8.06%	6.45%	11.63%	9.46%	10.71%	4.12%	5.26%

A total of 560 samples were collected from patients with HFMD and used for HEV detection during 2016 to 2022. There were 472 specimens (84.29%) that were positive for HEVs, including 16 with EV71 (2.86%), 102 with CA16 (18.21%), 296 with CA6 (52.86%), 14 with CA10 (2.50%) and 44 with other enteroviruses (7.86%) (Table 1). In 2018, the positivity rate of CA16 was higher than that of CA6, while in other years during 2016 to 2022, the CA6 positivity rate was significantly higher than that of other serotypes. EV71 was mainly detected between 2016 and 2017, with rates of 9.68 and 14.52%. There was only one sample positive for EV71 during 2021, with a rate of 1.03%. No EV71 was detected in 2018, 2019, 2020 or 2022.

We obtained 71 and 142 VP1 sequences from CA16 and CA6, respectively, from 2016 to 2022. Comparisons of the sequences showed that the 71 VP1 sequences from CA16 shared 78.8–100% identity at the nucleotide level, while the amino acid identity was 88.2–100%. The prototype of the CA16 VP1 sequence, G10 (U05876), was downloaded and showed 73.1–79.6% nucleotide identity and 89.2–96.9% amino acid identity with our CA16 sequences. Sequence comparisons showed that the 142 VP1 sequences from CA6 shared 62.1–100% nucleotide identity and 65.8–100% amino acid identity. The prototype of the CA6 VP1 sequence, Gdula (AY421764), was downloaded and showed 60.6–79.6% nucleotide identity and 65.4–96.9% amino acid identity with our CA6 sequences.

When compared with the prototype CA16 VP1 sequence, G10 (U05876), there were several amino acid substitutions, comprising T164K, I179V, L183M, A213E, P215L, S217A, N218D, A220L, I235V and V251I. N218D, A220L and V251I are considered to be associated with important neutralizing/antigenic epitopes within the VP1 protein that may be related to viral infectivity (14). Additionally, when compared with the prototype CA6 VP1 sequence, Gdula (AY421764), there were several amino acid substitutions, comprising P153R, V174I, N241D, V242I, H243Q, T277A, T283A, A299V, N300S, P301S, P301L, and S305Y. The corresponding sites and their properties have yet to be determined.

**Figure 3:**
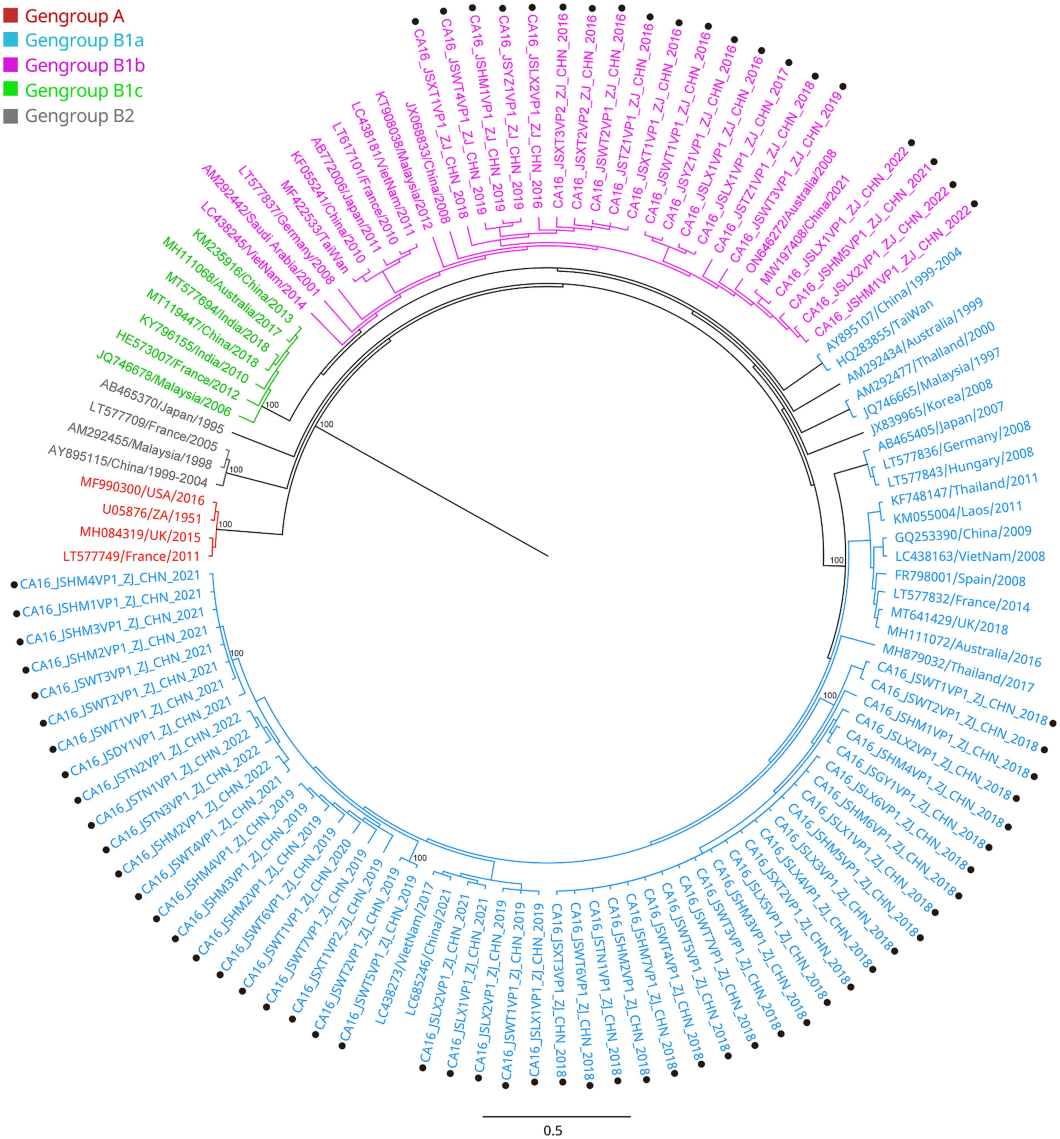
Phylogenetic analysis of CA16 based on VP1 region. Only 100% bootstrap values are shown. The clade in which the prototype of CA16 (U05876) was located was defined as the root. Sequences of CA16 genogroup A (the root) are indicated in red, genogroup B1a in light blue, genogroup B1b in purple, genogroup B1c in light green, genogroup B2 in dark. Sequences identified in the study are highlighted with black dots.

**Figure 4:**
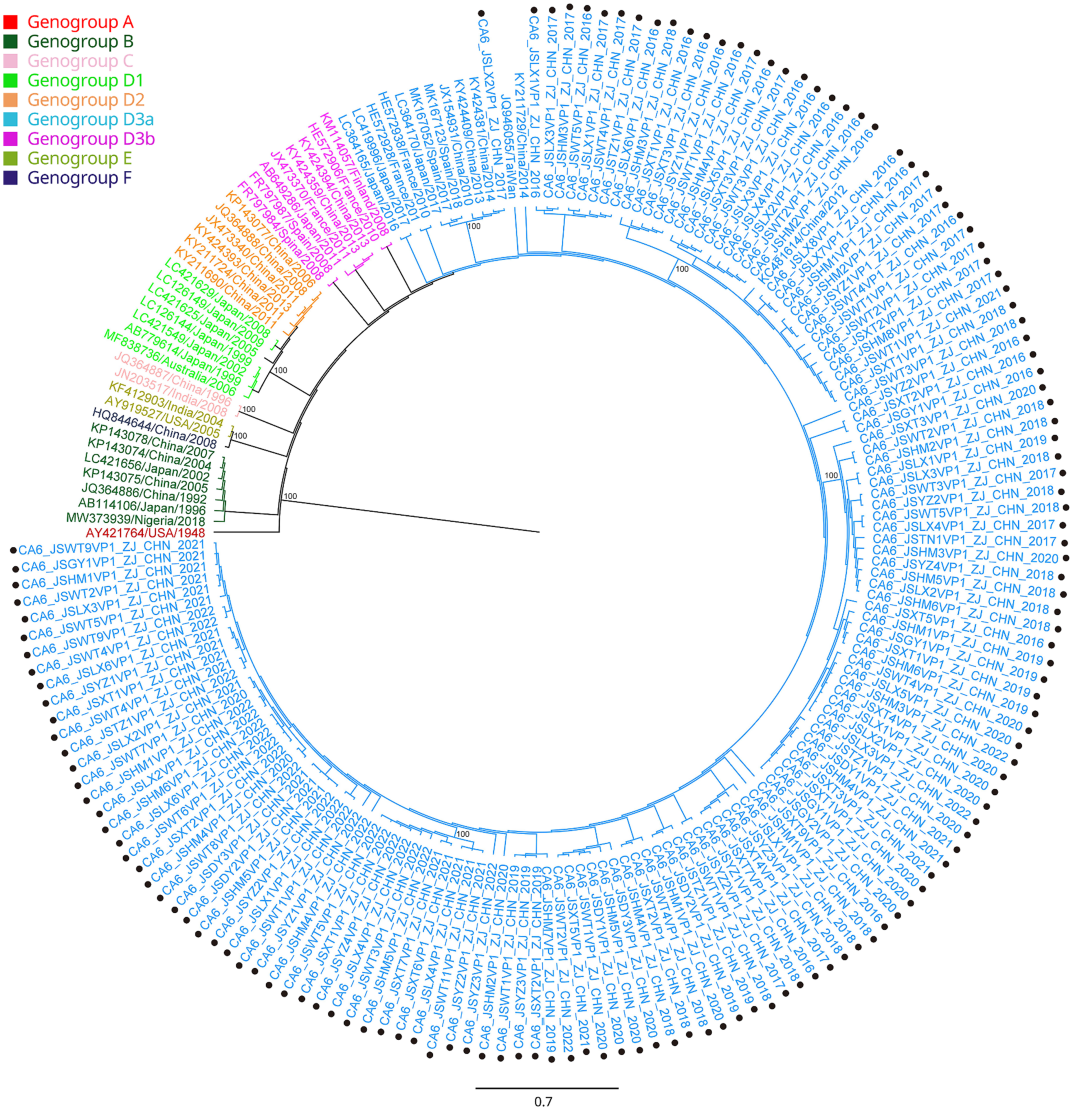
Phylogenetic analysis of CA6 based on VP1 region. Only 100% bootstrap values are shown. The clade in which the prototype of CA6 (AY421764) was located was defined as the root. Sequences of CA 6 genogroup A (the root) are indicated in red, genogroup B in dark green, genogroup C in light pink, genogroup D1 in light green, genogroup D2 in orange, genogroup D3a in light blue, genogroup D3b in purple, genogroup E in green and genogroup F in dark purple. Sequences identified in the study are highlighted with black dots.

All the VP1 sequences of CA16 were used to construct a phylogenetic tree with another 47 sequences of CA16, downloaded from GenBank, from different countries and years (Figure 3). CA16 was assigned to two genogroups (A and B) and formed a monophyletic clade. The prototype G10 strain (U05876) was in genogroup A. Genogroup B was split into B1 and B2. Genogroup B1 was separated into genogroups B1a, B1b and B1c. Sequences of CA16 were scattered among two different genogroups (B1a and B1b). The majority of sequences of CA16 were in the B1b genogroup (71.8%, 51/71). A phylogenetic tree of CA6 sequences was constructed with another 39 sequences, downloaded from GenBank, from different countries and years. The prototype Gdula (AY421764) was applied as the root sequence (Figure 4). CA6 was assigned to six genogroups and formed a monophyletic clade, comprising genogroups A – F. Genogroup D was split into D1, D2 and D3; the latter of which was further divided into D3a and D3b. Sequences of CA6 were assigned to the D3a genogroup.

## Discussion

Given its high infectivity, complex transmission route, rapid speed of spread and high susceptibility of children to the disease, HFMD became a notifiable infectious disease in 2008 after an outbreak in Anhui, China (15). EV71 and CA16 were generally considered to be the main pathogens causing HFMD in the past, and EV71 was the main pathogen associated with severe illness and deaths (16). Although the EV71 vaccine has been available in Zhejiang since 2016, the incidence of HFMD in the Jiashan area continues to fluctuate (Table 1). It was extremely high in 2018 and was lowest in 2021. Generally, the incidence of HFMD in Jiashan is seasonal, except in 2020 and 2022, which is consistent with other reports in China (12, 17). HFMD epidemics were sporadic from January to March each year, and the incidence began to increase in April, with a major epidemic peak from May to August, which declined in September, followed by a small secondary peak from October to December (Figure 1). Climatic factors, such as sunshine duration, temperature, precipitation and pressure, are considered to be important to seasonal epidemics of HFMD (18). However, the incidence during 2020 to 2022 was lower than in other years. One plausible explanation for this phenomenon was that the interventions introduced in response to coronavirus disease (COVID-19) facilitated the control of HFMD. Self-quarantine, social distancing, wearing face masks and frequent hand hygiene may have indirectly reduced the incidence of HFMD and prevented the outbreaks that occurred in other years (12, 19, 20). Strict and wide implementation of nonspecific nonpharmaceutical interventions, which differed from the relatively relaxed policies for COVID-19 during 2021, may have affected the endemic pattern of HEVs, leading to the unusual pattern of incidence in 2020 and 2022 (Figure 1). Additionally, it is likely that understaffing at health departments and decreased number of patients occurred during COVID-19 restrictions, which have been used to explain similar phenomena in other studies (19).

Human beings are usually considered to be the only reservoir of HEVs. Children aged 1–5 years comprised the majority of cases of HFMD in Jiashan, similar to other reports (11, 20). Children aged <1 year usually have maternal antibodies, while those aged 1–5 years have immature immune systems and undeveloped health habits when compared with older age groups. This age group should be targeted for HFMD control and prevention. In addition, more infections were found in urban areas than in the rural areas. The incidence of HFMD in urban areas of Jiashan, such as LX and WT, was higher than that in rural areas of Jiashan, such as TN, which is consistent with the explanation that socioeconomic factors play important roles in epidemics of HFMD (Figure 2). Generally, higher population density, mobility and frequent outdoor activities contribute to a higher risk of infections, and HFMD cases were mainly concentrated in cities (Figure 2) (21).

Another reason for the fluctuating incidence of HFMD after the introduction of EV71 vaccination is that there is no cross-protection between different serotypes of HEVs, and the co-circulation of multiple serotypes has become an epidemiological feature of HFMD outbreaks in Jiashan and other areas (22, 23). The HEV genogroups investigated in this study were consistent with circulating strains reported in mainland China in recent years (24, 25). From 2016 to 2022, CA16 and CA6 alternately became the predominant circulating strain in the Jiashan area. However, some CA10 and EV71 (re)-emerged alternately and sporadically during this period (Table 1). CA16 was the major pathogen associated with HFMD in the past. Since 2000, there has been a continuous epidemic of the B1 genogroup of CA16 in mainland China, with B1a dominating from 2008 to 2012 and B1b from 2013 to 2019, but with an increase in B1a since 2018 (24). Our results indicated that the main circulating CA16 genogroup between 2016 and 2017 was B1b (Figure 3). Genogroup B1a emerged in the Jiashan area in 2018 and became predominant during 2018 to 2022. No B1b genogroup CA16 were detected after 2017, indicating the need to strengthen molecular epidemiological surveillance. According to a previous study, HFMD had a peak incidence in Zhejiang Province in 2018 (12). Emergence of the B1a genogroup of CA16, a new genogroup in the Jiashan area, implies that lineage sorting for this serotype may be occurring in the Jiashan area. In contrast to CA16, D3a was the major genogroup of CA6 in circulation in Jiashan during 2016 to 2022, as indicated by our phylogenetic analysis, which is consistent with another study showing that D3a was predominant in China, with no indication of other emerging genogroups (Figure 4) (14). Control and prevention of CA6 might be more challenging than for CA16. First, the D3 genogroup has completed lineage sorting and is relatively genetically stable, which has led to outbreaks of HFMD worldwide, such as in Europe, Southeast Asia and mainland China (25). Second, the CA6 positivity rate was higher than that of CA16, EV71, CA10 and other HEVs in our study (Table 1). This suggests that more patients were infected with CA6 in the Jiashan area during 2016 to 2022, except for 2018. Other studies demonstrated similar data (19, 26). Lastly, CA6 is difficult to culture and mostly associated with mild cases of HFMD. It may be highly transmissible and therefore allow a smaller number of initially infected cases to significantly increase the incidence of infection (14, 26). Given its high transmissibility and mild virulence, CA6 might be underestimated when compared with other HEV serotypes. Therefore, a multivalent vaccine against HEVs other than EV71 (e.g., CA6, CA16, and CA10) needs to be developed in the near future.

In this study we reported the molecular epidemiologic pattern of HFMD and the genetic characteristics of different major serotypes of HEVs co-circulating in the Jiashan area during 2016 to 2022. Our results provide a scientific basis for future prevention, control and vaccine development for HFMD. Our study had some limitations. First, complete genomic information needs to be obtained for comprehensive understanding of the genetic features of different serotypes and their genogroups. Second, further detailed research on amino acid substitutions and their effects on virulence, antigenic shifts and genogroup dynamics of HEVs is required. Third, detailed analysis of the relationship between the genogroups identified in this study and the clinical course of the disease needs to be addressed. Continuous and comprehensive long-term surveillance for HFMD is needed to better understand and evaluate the prevalence and evolution of the corresponding pathogens, especially for the period after the introduction of the EV71 vaccine.

## Data availability statement

The datasets presented in this study can be found in online repositories. The names of the repository/repositories and accession number(s) can be found in the article/Supplementary material.

## Ethics statement

The studies involving humans were approved by Committee of Zhejiang Provincial Center for Disease Control and Prevention. The studies were conducted in accordance with the local legislation and institutional requirements. Written informed consent for participation in this study was provided by the participants' legal guardians/next of kin.

## Author contributions

YY: Formal analysis, Data curation, Methodology, Writing – original draft. YC: Writing – original draft, Investigation, Resources. JH: Investigation, Writing – original draft, Formal analysis. XB: Writing – original draft, Methodology. WS: Methodology, Writing – original draft. YS: Conceptualization, Formal analysis, Writing – review & editing. HM: Funding acquisition, Supervision, Writing – original draft.

## Footnotes

^1^ www.geneious.com

^2^ http://www.epidata.dk
