# Supplementary material for: Epidemiological and etiological investigations of hand, foot, and mouth disease in Jiashan, northeastern Zhejiang Province, China, during 2016 to 2022
Source: Front Public Health. 2024 May 1;12:1377861. doi: 10.3389/fpubh.2024.1377861 (PMC11094292; doi:10.3389/fpubh.2024.1377861)
Supplement: Supplementary file 1 [file Table_1.DOCX]

Supplementary Table 1 All sequences deposited in the GenBank database with accession number.

| Sequence Name | GenBank Accession Number |
| --- | --- |
| CA6_JSXT1VP1_ZJ_CHN_2019 | OR780127 |
| CA6_JSHM6VP1_ZJ_CHN_2019 | OR780128 |
| CA6_JSHM1VP1_ZJ_CHN_2019 | OR780129 |
| CA6_JSGY1VP1_ZJ_CHN_2019 | OR780130 |
| CA6_JSHM3VP1_ZJ_CHN_2020 | OR780131 |
| CA6_JSYZ4VP1_ZJ_CHN_2018 | OR780132 |
| CA6_JSLX2VP1_ZJ_CHN_2018 | OR780133 |
| CA6_JSHM6VP1_ZJ_CHN_2018 | OR780134 |
| CA6_JSHM5VP1_ZJ_CHN_2018 | OR780135 |
| CA6_JSXT5VP1_ZJ_CHN_2016 | OR780136 |
| CA6_JSHM4VP1_ZJ_CHN_2021 | OR780137 |
| CA6_JSLX5VP1_ZJ_CHN_2020 | OR780138 |
| CA6_JSHM3VP1_ZJ_CHN_2022 | OR780139 |
| CA6_JSXT4VP1_ZJ_CHN_2020 | OR780140 |
| CA6_JSLX1VP1_ZJ_CHN_2020 | OR780141 |
| CA6_JSWT4VP1_ZJ_CHN_2020 | OR780142 |
| CA6_JSLX2VP1_ZJ_CHN_2020 | OR780143 |
| CA6_JSLX3VP1_ZJ_CHN_2022 | OR780144 |
| CA6_JSXT3VP1_ZJ_CHN_2021 | OR780145 |
| CA6_JSXT1VP1_ZJ_CHN_2020 | OR780146 |
| CA6_JSGY1VP1_ZJ_CHN_2020 | OR780147 |
| CA6_JSGY2VP1_ZJ_CHN_2020 | OR780148 |
| CA6_JSDY1VP1_ZJ_CHN_2021 | OR780149 |
| CA6_JSTZ1VP1_ZJ_CHN_2020 | OR780150 |
| CA6_JSXT3VP1_ZJ_CHN_2020 | OR780151 |
| CA6_JSLX1VP1_ZJ_CHN_2019 | OR780152 |
| CA6_JSWT2VP1_ZJ_CHN_2018 | OR780153 |
| CA6_JSHM2VP1_ZJ_CHN_2018 | OR780154 |
| CA6_JSWT3VP1_ZJ_CHN_2017 | OR780155 |
| CA6_JSYZ2VP1_ZJ_CHN_2018 | OR780156 |
| CA6_JSLX3VP1_ZJ_CHN_2018 | OR780157 |
| CA6_JSWT5VP1_ZJ_CHN_2018 | OR780158 |
| CA6_JSLX4VP1_ZJ_CHN_2017 | OR780159 |
| CA6_JSTN1VP1_ZJ_CHN_2017 | OR780160 |
| CA6_JSLX3VP1_ZJ_CHN_2017 | OR780161 |
| CA6_JSHM3VP1_ZJ_CHN_2017 | OR780162 |
| CA6_JSLX1VP1_ZJ_CHN_2016 | OR780163 |
| CA6_JSTZ1VP1_ZJ_CHN_2017 | OR780164 |
| CA6_JSLX6VP1_ZJ_CHN_2016 | OR780165 |
| CA6_JSWT5VP1_ZJ_CHN_2016 | OR780166 |
| CA6_JSLX1VP1_ZJ_CHN_2017 | OR780167 |
| CA6_JSWT4VP1_ZJ_CHN_2017 | OR780168 |
| CA6_JSHM1VP1_ZJ_CHN_2018 | OR780169 |
| CA6_JSXT2VP1_ZJ_CHN_2016 | OR780170 |
| CA6_JSGY1VP1_ZJ_CHN_2016 | OR780171 |
| CA6_JSYZ2VP1_ZJ_CHN_2016 | OR780172 |
| CA6_JSXT9VP1_ZJ_CHN_2016 | OR780173 |
| CA6_JSYZ1VP1_ZJ_CHN_2018 | OR780174 |
| CA6_JSYZ3VP1_ZJ_CHN_2018 | OR780175 |
| CA6_JSXT7VP1_ZJ_CHN_2016 | OR780176 |
| CA6_JSXT4VP1_ZJ_CHN_2017 | OR780177 |
| CA6_JSLX1VP1_ZJ_CHN_2018 | OR780178 |
| CA6_JSWT1VP1_ZJ_CHN_2021 | OR780179 |
| CA6_JSLX7VP1_ZJ_CHN_2016 | OR780180 |
| CA6_JSHM1VP1_ZJ_CHN_2017 | OR780181 |
| CA6_JSWT1VP1_ZJ_CHN_2017 | OR780182 |
| CA6_JSWT2VP1_ZJ_CHN_2017 | OR780183 |
| CA6_JSXT2VP1_ZJ_CHN_2017 | OR780184 |
| CA6_JSXT1VP1_ZJ_CHN_2018 | OR780185 |
| CA6_JSWT3VP1_ZJ_CHN_2018 | OR780186 |
| CA6_JSHM8VP1_ZJ_CHN_2017 | OR780187 |
| CA6_JSYZ1VP1_ZJ_CHN_2017 | OR780188 |
| CA6_JSWT4VP1_ZJ_CHN_2016 | OR780189 |
| CA6_JSHM2VP1_ZJ_CHN_2017 | OR780190 |
| CA6_JSXT1VP1_ZJ_CHN_2016 | OR780191 |
| CA6_JSYZ1VP1_ZJ_CHN_2016 | OR780192 |
| CA6_JSXT3VP1_ZJ_CHN_2016 | OR780193 |
| CA6_JSWT1VP1_ZJ_CHN_2016 | OR780194 |
| CA6_JSLX8VP1_ZJ_CHN_2016 | OR780195 |
| CA6_JSHM3VP1_ZJ_CHN_2018 | OR780196 |
| CA6_JSLX3VP1_ZJ_CHN_2016 | OR780197 |
| CA6_JSLX4VP1_ZJ_CHN_2016 | OR780198 |
| CA6_JSWT2VP1_ZJ_CHN_2016 | OR780199 |
| CA6_JSHM2VP1_ZJ_CHN_2016 | OR780200 |
| CA6_JSLX2VP1_ZJ_CHN_2016 | OR780201 |
| CA6_JSHM4VP1_ZJ_CHN_2017 | OR780202 |
| CA6_JSXT3VP1_ZJ_CHN_2017 | OR780203 |
| CA6_JSLX5VP1_ZJ_CHN_2016 | OR780204 |
| CA6_JSWT3VP1_ZJ_CHN_2016 | OR780205 |
| CA6_JSLX2VP1_ZJ_CHN_2017 | OR780206 |
| CA6_JSXT2VP1_ZJ_CHN_2022 | OR780207 |
| CA6_JSWT6VP1_ZJ_CHN_2022 | OR780208 |
| CA6_JSLX6VP1_ZJ_CHN_2022 | OR780209 |
| CA6_JSHM6VP1_ZJ_CHN_2022 | OR780210 |
| CA6_JSYZ1VP1_ZJ_CHN_2022 | OR780211 |
| CA6_JSWT4VP1_ZJ_CHN_2022 | OR780212 |
| CA6_JSWT8VP1_ZJ_CHN_2022 | OR780213 |
| CA6_JSWT5VP1_ZJ_CHN_2021 | OR780214 |
| CA6_JSLX3VP1_ZJ_CHN_2021 | OR780215 |
| CA6_JSWT2VP1_ZJ_CHN_2021 | OR780216 |
| CA6_JSHM1VP1_ZJ_CHN_2021 | OR780217 |
| CA6_JSGY1VP1_ZJ_CHN_2021 | OR780218 |
| CA6_JSWT9VP1_ZJ_CHN_2021 | OR780219 |
| CA6_JSWT9VP1_ZJ_CHN_2022 | OR780220 |
| CA6_JSHM4VP1_ZJ_CHN_2020 | OR780221 |
| CA6_JSWT7VP1_ZJ_CHN_2020 | OR780222 |
| CA6_JSLX2VP1_ZJ_CHN_2021 | OR780223 |
| CA6_JSXT1VP1_ZJ_CHN_2021 | OR780224 |
| CA6_JSYZ1VP1_ZJ_CHN_2021 | OR780225 |
| CA6_JSLX6VP1_ZJ_CHN_2021 | OR780226 |
| CA6_JSLX2VP1_ZJ_CHN_2022 | OR780227 |
| CA6_JSHM1VP1_ZJ_CHN_2022 | OR780228 |
| CA6_JSTZ1VP1_ZJ_CHN_2022 | OR780229 |
| CA6_JSWT4VP1_ZJ_CHN_2021 | OR780230 |
| CA6_JSWT1VP1_ZJ_CHN_2022 | OR780231 |
| CA6_JSLX1VP1_ZJ_CHN_2022 | OR780232 |
| CA6_JSYZ2VP1_ZJ_CHN_2022 | OR780233 |
| CA6_JSHM5VP1_ZJ_CHN_2021 | OR780234 |
| CA6_JSDY2VP1_ZJ_CHN_2021 | OR780235 |
| CA6_JSDY3VP1_ZJ_CHN_2021 | OR780236 |
| CA6_JSYZ2VP1_ZJ_CHN_2017 | OR780237 |
| CA6_JSWT1VP1_ZJ_CHN_2018 | OR780238 |
| CA6_JSYZ3VP1_ZJ_CHN_2022 | OR780239 |
| CA6_JSXT6VP1_ZJ_CHN_2021 | OR780240 |
| CA6_JSXT7VP1_ZJ_CHN_2021 | OR780241 |
| CA6_JSYZ2VP1_ZJ_CHN_2021 | OR780242 |
| CA6_JSWT11VP1_ZJ_CHN_2021 | OR780243 |
| CA6_JSLX4VP1_ZJ_CHN_2021 | OR780244 |
| CA6_JSHM4VP1_ZJ_CHN_2022 | OR780245 |
| CA6_JSLX4VP1_ZJ_CHN_2022 | OR780246 |
| CA6_JSYZ4VP1_ZJ_CHN_2022 | OR780247 |
| CA6_JSXT1VP1_ZJ_CHN_2022 | OR780248 |
| CA6_JSWT5VP1_ZJ_CHN_2022 | OR780249 |
| CA6_JSHM5VP1_ZJ_CHN_2022 | OR780250 |
| CA6_JSWT3VP1_ZJ_CHN_2022 | OR780251 |
| CA6_JSTZ1VP1_ZJ_CHN_2019 | OR780252 |
| CA6_JSDY2VP1_ZJ_CHN_2019 | OR780253 |
| CA6_JSHM1VP1_ZJ_CHN_2020 | OR780254 |
| CA6_JSXT5VP1_ZJ_CHN_2021 | OR780255 |
| CA6_JSDY3VP1_ZJ_CHN_2020 | OR780256 |
| CA6_JSWT2VP1_ZJ_CHN_2022 | OR780257 |
| CA6_JSHM5VP1_ZJ_CHN_2020 | OR780258 |
| CA6_JSDY1VP1_ZJ_CHN_2020 | OR780259 |
| CA6_JSWT1VP1_ZJ_CHN_2020 | OR780260 |
| CA6_JSWT4VP1_ZJ_CHN_2018 | OR780261 |
| CA6_JSHM2VP1_ZJ_CHN_2020 | OR780262 |
| CA6_JSXT2VP1_ZJ_CHN_2019 | OR780263 |
| CA6_JSYZ3VP1_ZJ_CHN_2019 | OR780264 |
| CA6_JSWT1VP1_ZJ_CHN_2019 | OR780265 |
| CA6_JSHM7VP1_ZJ_CHN_2019 | OR780266 |
| CA6_JSXT2VP1_ZJ_CHN_2018 | OR780267 |
| CA6_JSHM4VP1_ZJ_CHN_2018 | OR780268 |
| CA16_JSWT3VP1_ZJ_CHN_2019 | OR780269 |
| CA16_JSLX1VP1_ZJ_CHN_2022 | OR780270 |
| CA16_JSLX2VP1_ZJ_CHN_2022 | OR780271 |
| CA16_JSHM1VP1_ZJ_CHN_2022 | OR780272 |
| CA16_JSHM5VP1_ZJ_CHN_2021 | OR780273 |
| CA16_JSTZ1VP1_ZJ_CHN_2018 | OR780274 |
| CA16_JSXT1VP1_ZJ_CHN_2018 | OR780275 |
| CA16_JSLX1VP1_ZJ_CHN_2016 | OR780276 |
| CA16_JSLX1VP1_ZJ_CHN_2017 | OR780277 |
| CA16_JSWT1VP1_ZJ_CHN_2016 | OR780278 |
| CA16_JSYZ1VP1_ZJ_CHN_2016 | OR780279 |
| CA16_JSHM1VP1_ZJ_CHN_2019 | OR780280 |
| CA16_JSYZ1VP1_ZJ_CHN_2019 | OR780281 |
| CA16_JSWT4VP1_ZJ_CHN_2019 | OR780282 |
| CA16_JSLX2VP1_ZJ_CHN_2016 | OR780283 |
| CA16_JSWT2VP1_ZJ_CHN_2016 | OR780284 |
| CA16_JSTZ1VP1_ZJ_CHN_2016 | OR780285 |
| CA16_JSXT1VP1_ZJ_CHN_2016 | OR780286 |
| CA16_JSXT2VP2_ZJ_CHN_2016 | OR780287 |
| CA16_JSXT3VP2_ZJ_CHN_2016 | OR780288 |
| CA16_JSLX1VP1_ZJ_CHN_2019 | OR780289 |
| CA16_JSWT1VP1_ZJ_CHN_2019 | OR780290 |
| CA16_JSLX2VP1_ZJ_CHN_2019 | OR780291 |
| CA16_JSWT1VP1_ZJ_CHN_2018 | OR780292 |
| CA16_JSWT2VP1_ZJ_CHN_2018 | OR780293 |
| CA16_JSGY1VP1_ZJ_CHN_2018 | OR780294 |
| CA16_JSHM1VP1_ZJ_CHN_2018 | OR780295 |
| CA16_JSHM4VP1_ZJ_CHN_2018 | OR780296 |
| CA16_JSLX2VP1_ZJ_CHN_2018 | OR780297 |
| CA16_JSLX6VP1_ZJ_CHN_2018 | OR780298 |
| CA16_JSHM6VP1_ZJ_CHN_2018 | OR780299 |
| CA16_JSLX1VP1_ZJ_CHN_2018 | OR780300 |
| CA16_JSHM5VP1_ZJ_CHN_2018 | OR780301 |
| CA16_JSLX3VP1_ZJ_CHN_2018 | OR780302 |
| CA16_JSLX4VP1_ZJ_CHN_2018 | OR780303 |
| CA16_JSXT2VP1_ZJ_CHN_2018 | OR780304 |
| CA16_JSLX5VP1_ZJ_CHN_2018 | OR780305 |
| CA16_JSHM3VP1_ZJ_CHN_2018 | OR780306 |
| CA16_JSWT3VP1_ZJ_CHN_2018 | OR780307 |
| CA16_JSWT7VP1_ZJ_CHN_2018 | OR780308 |
| CA16_JSWT5VP1_ZJ_CHN_2018 | OR780309 |
| CA16_JSWT4VP1_ZJ_CHN_2018 | OR780310 |
| CA16_JSHM7VP1_ZJ_CHN_2018 | OR780311 |
| CA16_JSHM2VP1_ZJ_CHN_2018 | OR780312 |
| CA16_JSTN1VP1_ZJ_CHN_2018 | OR780313 |
| CA16_JSWT6VP1_ZJ_CHN_2018 | OR780314 |
| CA16_JSXT3VP1_ZJ_CHN_2018 | OR780315 |
| CA16_JSWT1VP1_ZJ_CHN_2021 | OR780316 |
| CA16_JSWT2VP1_ZJ_CHN_2021 | OR780317 |
| CA16_JSWT3VP1_ZJ_CHN_2021 | OR780318 |
| CA16_JSHM2VP1_ZJ_CHN_2021 | OR780319 |
| CA16_JSHM3VP1_ZJ_CHN_2021 | OR780320 |
| CA16_JSDY1VP1_ZJ_CHN_2021 | OR780321 |
| CA16_JSHM1VP1_ZJ_CHN_2021 | OR780322 |
| CA16_JSHM4VP1_ZJ_CHN_2021 | OR780323 |
| CA16_JSHM2VP1_ZJ_CHN_2022 | OR780324 |
| CA16_JSWT4VP1_ZJ_CHN_2021 | OR780325 |
| CA16_JSTN3VP1_ZJ_CHN_2022 | OR780326 |
| CA16_JSTN1VP1_ZJ_CHN_2022 | OR780327 |
| CA16_JSTN2VP1_ZJ_CHN_2022 | OR780328 |
| CA16_JSLX1VP1_ZJ_CHN_2021 | OR780329 |
| CA16_JSLX2VP1_ZJ_CHN_2021 | OR780330 |
| CA16_JSWT6VP1_ZJ_CHN_2019 | OR780331 |
| CA16_JSHM2VP1_ZJ_CHN_2019 | OR780332 |
| CA16_JSHM3VP1_ZJ_CHN_2019 | OR780333 |
| CA16_JSHM4VP1_ZJ_CHN_2019 | OR780334 |
| CA16_JSWT2VP1_ZJ_CHN_2019 | OR780335 |
| CA16_JSWT5VP1_ZJ_CHN_2019 | OR780336 |
| CA16_JSWT1VP1_ZJ_CHN_2020 | OR780337 |
| CA16_JSWT7VP1_ZJ_CHN_2019 | OR780338 |
| CA16_JSXT1VP2_ZJ_CHN_2019 | OR780339 |
